# Increased Circulating Exosomal miRNA-223 Is Associated with Acute Ischemic Stroke

**DOI:** 10.3389/fneur.2017.00057

**Published:** 2017-02-27

**Authors:** Yajing Chen, Yaying Song, Jun Huang, Meijie Qu, Yu Zhang, Jieli Geng, Zhijun Zhang, Jianrong Liu, Guo-Yuan Yang

**Affiliations:** ^1^Department of Neurology, Ruijin Hospital and Ruijin Hospital North, Shanghai Jiao Tong University School of Medicine, Shanghai, China; ^2^Department of Neurology, Shanghai General Hospital, Shanghai Jiao Tong University School of Medicine, Shanghai, China; ^3^Neuroscience and Neuroengineering Center, School of Biomedical Engineering, Med-X Research Institute, Shanghai Jiao Tong University, Shanghai, China; ^4^Shanghai Key Laboratory of Hypertension, Department of Hypertension, Ruijin Hospital, Shanghai Institute of Hypertension, Shanghai Jiao Tong University School of Medicine, Shanghai, China; ^5^Department of Neurology, Renji Hospital, Shanghai Jiao Tong University School of Medicine, Shanghai, China

**Keywords:** acute ischemic stroke, biomarker, blood, diagnosis, exosomes, humans, microRNA, prognostic

## Abstract

Recent studies have demonstrated that exosomal microRNAs (miRNAs) are novel biomarkers and therapeutic targets for various diseases including vascular disease. However, specific exosomal miRNAs expression in stroke patients has not been reported yet. Here, we explored whether circulating exosomal miRNAs can serve as potential biomarkers for the diagnosis of acute ischemic stroke and discussed the potential for clinical application. Blood samples were collected from acute ischemic stroke patients within the first 72 h (*n* = 50). Circulating exosomes were exacted by Exoquick exosome isolation kit and characterized by transmission electron microscopy. Western blot was performed to assess the expression of exosomal protein makers. Exosomal miRNA-223 (miR-223) was detected by RT-PCR assay. The relationship between the expression levels of miR-223 and National Institutes of Health Stroke Scale (NIHSS) scores, brain infarct volume, and neurological outcomes were analyzed. Circulating exosomes were isolated and the size of vesicles ranged between 30 and 100 nm. The identification of exosomes was further confirmed by the detection of specific exosomal protein markers CD9, CD63, and Tsg101. Exosomal miR-223 in acute ischemic stroke patients was significantly upregulated compared to control group (*p* < 0.001). Exosomal miR-223 level was positively correlated with NIHSS scores (*r* = 0.31, *p* = 0.03). Exosomal miR-223 expression in stroke patients with poor outcomes was higher than those with good outcomes (*p* < 0.05). Increased exosomal miR-223 was associated with acute ischemic stroke occurrence, stroke severity, and short-term outcomes. Future studies with large sample are needed to assess the clinical application of exosomal miR-223 as a novel biomarker for ischemic stroke diagnosis.

## Introduction

Stroke is the second leading cause of death in the worldwide, bringing heavy burden to the society and families (1). Although the morbidity and mortality have been reduced in high-income countries due to the public stroke education and the control of conventional stroke risk factors, the molecular mechanism of the pathogenesis of ischemic stroke still remains unclear.

Generally, the diagnosis of stroke relies on the clinical manifestation of the suspected patients and later confirmation by Computed Tomography (CT) or MR imaging examination. However, it is sometimes difficult for the physicians to make objective judgment when the symptom is slight or hard to differentiate from other neurological and non-neurological diseases. The limitation of brain imaging system in detecting small infarcts should also be noticed. Therefore, abundant studies on biomarkers have been carried out to provide ideal diagnosis for stroke (2–4).

Animal and *in vitro* experiments demonstrated that microRNAs (miRNAs) were involved greatly in stroke pathogenesis by regulating oxide stress, inflammation, apoptosis, and endothelial function (5–8). Clinical studies also proved that circulating miRNAs were potential biomarkers, which could facilitate the diagnosis of stroke, the identification of subtype, the evaluation of severity, and the prediction of prognosis (9–14).

Exosomes are 30–100 nm vesicles that cells secrete into extracellular space when multivesicular bodies fuse with cell membrane. Initially, exosomes were regarded as cell garbages to remove unwanted proteins. It was not until 2007, when Valadi et al. first identified the existence of RNA and miRNA that important role of exosomes was realized—a novel carrier for intercellular genetic material exchange and communication (15). In recent years, abundant studies provided strong evidence that exosomal miRNAs were novel biomarkers and therapy targets for various diseases such as tumor, neurodegeneration disease, and vascular disease (16–22). However, whether exosomal miRNAs can serve as biomarkers for stroke diagnosis remains largely unknown.

microRNA-223 (miR-223) was shown to be one of the most highly expressed miRNAs in plasma exosomes of healthy human and results about its function in ischemia injury are inconsistent (23). Here, we designed a retrospective case–control study to detect the level of exosomal miR-223 expression in stroke patients and non-stroke subjects. We then analyzed the association between exosomal miR-223 expression and National Institutes of Health Stroke Scale (NIHSS) scores, infarct volume, and modified Rankin Scale (mRS) in stroke patients. We also discussed the potential application of exosomes being novel approach for stroke diagnosis and therapy.

## Materials and Methods

### Subject and Sample Collection

Human project was approved by the committee of Institutional Review Board (IRB) of Shanghai Jiao Tong University, Shanghai, China. Written informed consent was obtained from patients according to the Helsinki Declaration, and the Helsinki Declaration was followed during the human studies. Peripheral blood was collected from stroke patients who were recruited consecutively from September 2014 to June 2015 in Ruijin Hospital. Control group were enrolled in January 2015 from subjects who came for medical examination and denied previous history of stroke attack. Subjects in each group were matched with age and gender. Demography feature, related previous history including hypertension, diabetes mellitus (DM), hyperlipidemia, cardiopathy, associated laboratory test, and imaging information including blood glucose, blood lipid, electrocardiogram, cardiac ultrasonography, carotid artery ultrasonography, MR imaging, and MR angiography were also collected for analysis. The risk factors were defined as the following: (1) hypertension: blood pressure ≥140/90 mmHg; (2) DM: fasting glucose ≥7.0 mmol/L or 2-h postprandial blood glucose ≥11.1 mmol/L; (3) hyperlipidemia: triglycerides >1.7 mmol/L or total cholesterol >5.7 mmol/L or low-density lipoprotein >4.3 mmol/L. The exclusive criteria included recurrent stroke or stroke onset longer than 72 h, renal or liver failure, acute infectious disease, tumor, hematologic disease, and patients who are unable to cooperate with physical examination.

The severity of stroke was evaluated with NIHSS scores. The patients were classified into three groups: large artery atherosclerotic stroke (LA), cardioembolism (CE), and small artery stroke (SA) according to the Trial of Org 10172 in Acute Stroke Treatment (TOAST) classification. Patients were also classified into the following four groups: total anterior circulation infarct, partial anterior circulation infarct (PACI), posterior circulation infarct (POCI), and lacunar infarct (LACI) according to Oxfordshire Community Stroke Project (OCSP) definition. The infarct volume was calculated by ABC/2 method (A and B represent the largest diameter of the infarct and its largest perpendicular diameter, while C represents the thickness of the slices where the infarct lesion was visible). 3-month mRS was used to assess the neurologic outcome of stroke patients.

### Blood Preparation and Exosomes Isolation

Whole blood was drawn into promoting coagulating tubes and centrifuged at 1,600 *g* for 10 min. Serum was aspirated and stored at −80°C. ExoQuick exosome precipitation solution (System Biosciences, CA, USA) was used to precipitate serum exosomes. Briefly, centrifuged the serum sample at 3,000 *g* for 15 min to remove cell debris; Then, added 125 μL ExoQuick exosome precipitation solution to 500 μL serum and mixed well; after refrigerating at 4°C for 30 min, centrifuged the mixture at 1,500 *g* for 30 min and removed the supernatant; Centrifuged another 5 min to spin down residual fluid and resuspended exosomes pellet by PBS or nuclease-free water. All centrifugations were done at 4°C.

### Exosomes Identification

The size and morphology feature of exosomes were examined by transmission electron microscopy (TEM) (TEM, Phillip CM120, 60 kV). Protein were extracted from exosomes resuspension solution with RIPA lysis buffer and separated on 8% polyacrylamide gel before transferring to a nitrocellulose filter membrane. The blotting membrane was blocked with skim milk and incubated with CD9 antibody (1:1,000 dilution, Abcam, Cambridge, UK)/CD63 antibody (1:200 dilution, Santa Cruz Biotechnology Inc., Santa Cruz, CA, USA)/TSG101 (1:500 dilution, Abcam, Cambridge, UK) overnight. After TBST washing, secondary HRP-conjugated antibody was added for 1 h (1:5,000 dilution, HuaAn Biotechnology Inc., Hangzhou, China). The proteins were finally detected using chemiluminescence (Thermo Scientific, Rockford, IL, USA).

### RNA Extraction and Reverse Transcription

Total RNA was isolated from exosomes using miRNeasy Mini Kit (Qiagen, Hilden, Germany) mainly according to the manufacturer’s instructions. The qiazol volume was 700 µl, and the final elute volume was 20 µl. Single-strand cDNA was synthesized using universal cDNA synthesis kit (Exiqon, Vedbaek, Denmark) following the condition: 42°C for 1 h and then 95°C for 5 min.

### Real-time PCR

The expression of miRNA was tested by real-time PCR using SYBR Green master mix (Exiqon, Vedbaek, Denmark) on an Applied Biosystems 7900 real-time PCR machine (Life Technologies). The reaction conditions were as follows: 95°C for 10 min followed by 40 cycles of 95°C for 10 s and 60°C for 1 min. The relative expression of miR-223 was normalized to the endogenous control miR-16 expression using the comparative cycle threshold (CT) method. When comparing different groups, the data were logarithm-transformed.

### Statistical Analysis

Statistics were calculated using SPSS 19 (IBM SPSS Inc., NY, USA). The Kolmogorov–Smirnov test was used to determine the normality of continuous variables distribution. If normal, Student’s *t*-test was used to compare differences between groups, if abnormal, Welch correction was used. Proportions were compared using the chi-square test or Fisher’s test.

To test the feasibility of using miR-223 as a biomarker for stroke, we built statistical model by multivariable logistic regression analysis. Forward stepwise selection procedures were adopted. Results were expressed as adjusted odds ratios (OR) with the corresponding 95% confidence intervals (95% CI). Correlations were estimated by Pearson correlation test. Differences were considered significant at *p* < 0.05.

## Results

### The Identification of Circulating Exosomes

**Figure 1:**
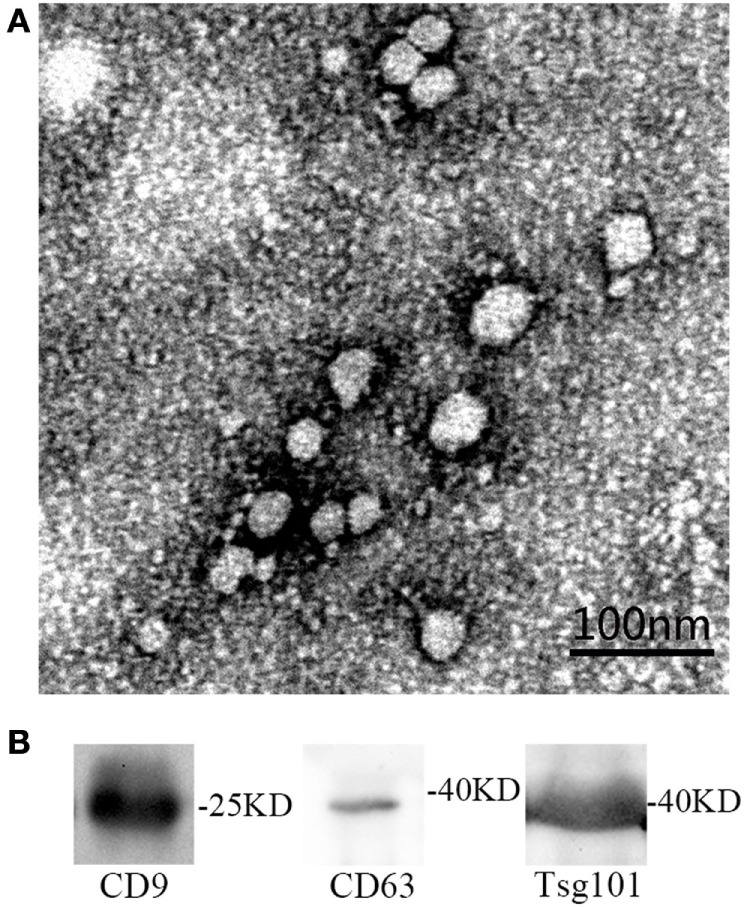
**(A)** The identification of serum exosomes by transmission electron microscope. **(B)** The identification of exosomes specific proteins by western blot.

Transmission electron microscopy and western blot were used for identification of circulating exosomes. On the one hand, the yielded vesicles were consistent with exosomes in size and morphology—30–100 nm nano-size and round-shaped (Figure 1A). Some researchers showed TEM picture of cup-shaped exosomes (24). The shape difference is caused by different methods of preparing sample (25). On the other hand, western blot proved the expression of certain exosomal marker proteins, i.e., CD9, CD63, and Tsg101 (Figure 1B). CD9 and CD63 are the most commonly used marker proteins for exosomes identification. Tsg101 involves in transmembrane cargo inclusion and is important for exosomes biogenesis (26).

### Baseline Clinical Characteristics

The clinical characteristics of ischemic stroke patients (*n* = 50) and control subjects (*n* = 33) are shown in Table 1. Mean age and the distribution of race/ethnicity were not different between two groups (*p* = 0.67, 1, 1). The number of male in both groups was about 1.5 times that of female (*p* = 0.75). A greater percentage of risk factors in stroke patients was found including DM 36.0% (*p* < 0.05) and cardiopathy 30.0% (*p* < 0.05). In our study, stroke patients and control group had the similar percentage of hypertension (*p* = 0.98). We considered that this phenomenon may be due to the increased awareness of health care in people with hypertension. They were more willing to participate in our research. The mean NIHSS score of stroke patients was relatively low (3.32). According to the TOAST classification, 50.0% of patients were classified as LA, while cardioembolism 16.0% and SA 34.0%. A smaller percentage of cardioembolism subtype is because that these patients were mostly enrolled to the Department of Cardiology in our hospital. According to the OCSP classification, 22 stroke patients were classified as PACI, while POCI subgroup was 16 cases and LACI subgroup was 11 cases. Patients with good outcome (mRS ≤ 2) took up 60% and those with poor outcome (mRS > 2) accounted for 40%.

**Table 1:** **Clinical characteristics of ischemic stroke patients and healthy control**.

		Stroke	Control	*p*-Value
Total (*N*)		50	33	NA
Race (Asian, %)		100%	100%	1
Ethnicity (Han, %)		100%	100%	1
Age (years, mean ± SD)		64 ± 9.4	63 ± 7.9	0.67
Sex (male/female, *N*)		32/18	20/13	0.75
Hypertension (*N*, %)		38 (76.0%)	25 (75.8%)	0.98
Hyperlipidemia (*N*, %)		29 (58.0%)	14 (42.4%)	0.17
Diabetes mellitus (DM) (*N*, %)		18 (36.0%)	3 (9.1%)	<0.05
Cardiopathy (*N*, %)		15 (30.0%)	1 (3.0%)	<0.05
NIHSS (mean, min, max)		3.32 (0.14)	NA	NA
TOAST	LA	25 (50.0%)	NA	NA
	CE	8 (16.0%)	NA	NA
	SA	17 (34.0%)	NA	NA
OCSP	TACI	1 (2.0%)	NA	NA
	PACI	22 (44.0%)	NA	NA
	Posterior circulation infarct	16 (32.0%)	NA	NA
	LACI	11 (22.0%)	NA	NA
mRS	≤2	30 (60.0%)	NA	NA
	>2	20 (40.0%)	NA	NA

*The difference of age between stroke (*n* = 50) and control group (*n* = 33) were compared with Student’s *t*-test. The proportions of race, sex, hypertension, hyperlipidemia, DM, and cardiopathy between two groups were compared with chi-square test*.

*NIHSS, National Institutes of Health Stroke Scale; TOAST, Trial of Org 10172 in Acute Stroke Treatment; LA, large artery atherosclerotic stroke; CE, cardioembolism; SA, small artery stroke; OCSP, Oxfordshire Community Stroke Project; TACI, total anterior circulation infarct; PACI, partial anterior circulation infarct; LACI, lacunar infarct; mRS, modified Rankin Scale; NA, not available*.

### Exosomal miR-223 Expression in Acute Ischemic Stroke Patients

**Figure 2:**
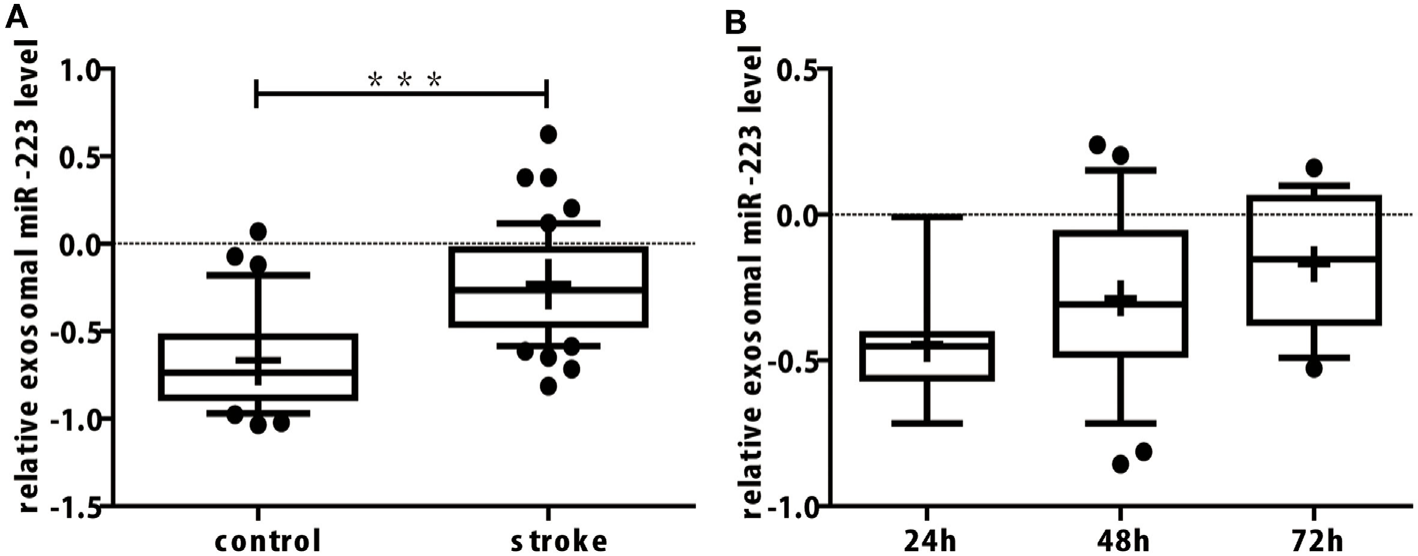
**(A)** The expression of miR-223 in serum exosomes of acute ischemic stroke patients was upregulated significantly. The difference of exosomal miR-223 level between stroke and control group were compared with Student’s *t*-test. Box plots showed the expression of exosomal miR-223 in patients and control. The *y*-axis indicated miR-223 expression levels by log10 change. ***p < 0.001, stroke patients vs. control. Data are median (25% percentile, 75% percentile), *n* = 50 in stroke group, *n* = 33 in control group. **(B)** Serum exosomal miR-223 expression of stroke patients between different onset time. Exosomal miR-223 levels of different onset times were compared with one-way ANOVA analysis. Box plots showed the expression of exosomal miR-223 in stroke patients with different onset times. The *y*-axis indicated miR-223 expression levels by log10 change. Data are median (25% percentile, 75% percentile), *n* = 7 in 24 h subgroup, *n* = 25 in 48 h subgroup, and *n* = 18 in 72 h subgroup.

In our study, miR-16 was used as internal control. Exosomal miR-16 level was almost the same between the two groups (Table 2, *p* = 0.828). When compared to control group, the expression of exosomal miR-223 in the serum of patients with acute ischemic stroke were increased greatly (Table 2; Figure 2A, *p* < 0.001). To explore the changing tendency of miR-223 after stroke attack, we analyzed circulating exosomal miR-223 levels at 24, 48, and 72 h. Exosomal miR-223 expression was not significantly different between subgroups, although we found it had a tendency to increase by time (Figure 2B).

**Table 2:** **Exosomal miR-16 and miR-223 levels of ischemic stroke patients and healthy control**.

	Stroke	Control	*p* Value
miR-16 CT value (mean ± SD)	21.88 ± 1.18	21.82 ± 1.51	0.828
miR-223 expression (mean ± SD)	−0.23 ± 0.30	−0.67 ± 0.28	<0.001

*Student’s *t*-test was used to compare exosomal miR-16/miR-223 levels of stroke patients (*n* = 50) and healthy control (*n* = 33)*.

*CT, cycle threshold*.

### The Relationship between Exosomal miR-223 Expression and Stroke Onset

**Figure 3:**
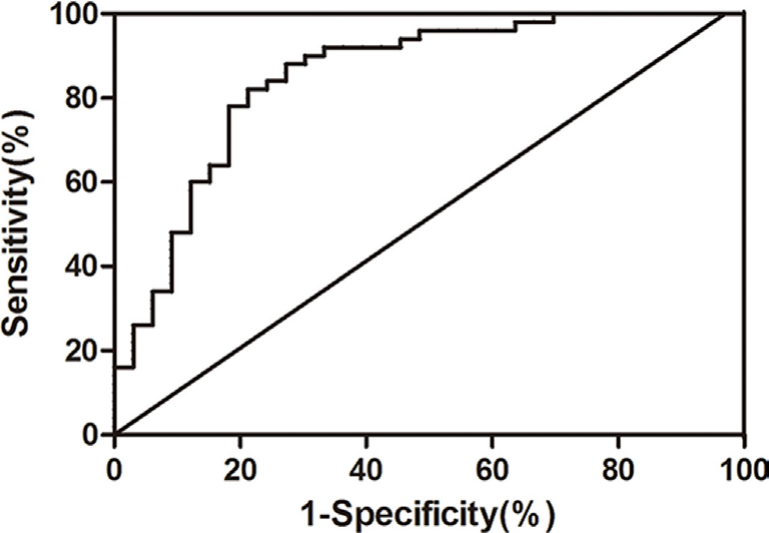
**The receiver operating characteristic curve of serum exosomal miR-223 diagnosing ischemic stroke**.

To eliminate the unmatched risk factors impact (DM and cardiopathy) on exosomal miR-223 expression between two groups, we performed a logistic regression analysis. The result indicated that circulating exosomal miR-223 expression was a possible risk factor of stroke (Table 3, *p* < 0.001). In addition, when the value of exosomal miR-223 increased by 0.1, the risk of stroke occurrence increased by about 0.7-fold. The receiver operating characteristic curve was drawn to evaluate the diagnostic value of exosomal miR-223 (Figure 3). The area under the curve was 0.859, when circulating exosomal miR-223 = −0.50, had the best sensitivity (84.0%) and specificity (78.8%) for stroke diagnosis.

**Table 3:** **Exosomal miR-223 expression and the risk of stroke**.

Factors	Adjusted *p*-value	OR	95% CI
miR-223	<0.001	1.70	1.31–2.20
Diabetes mellitus (DM)	0.015	7.84	1.50–40.98
Cardiopathy	0.073	7.72	0.83–72.20

*Exosomal miR-223 expression together with conventional stroke risks such as hypertension, hyperlipidemia, DM, and cardiopathy were analyzed in a statistical model by multivariable logistic regression*.

*OR, odds ratios; CI, confidence intervals*.

### The Relationship between the Expression of Exosomal miR-223 and Characteristics of TOAST Subtypes, NIHSS Score, Infarct Volume, and mRS

**Figure 4:**
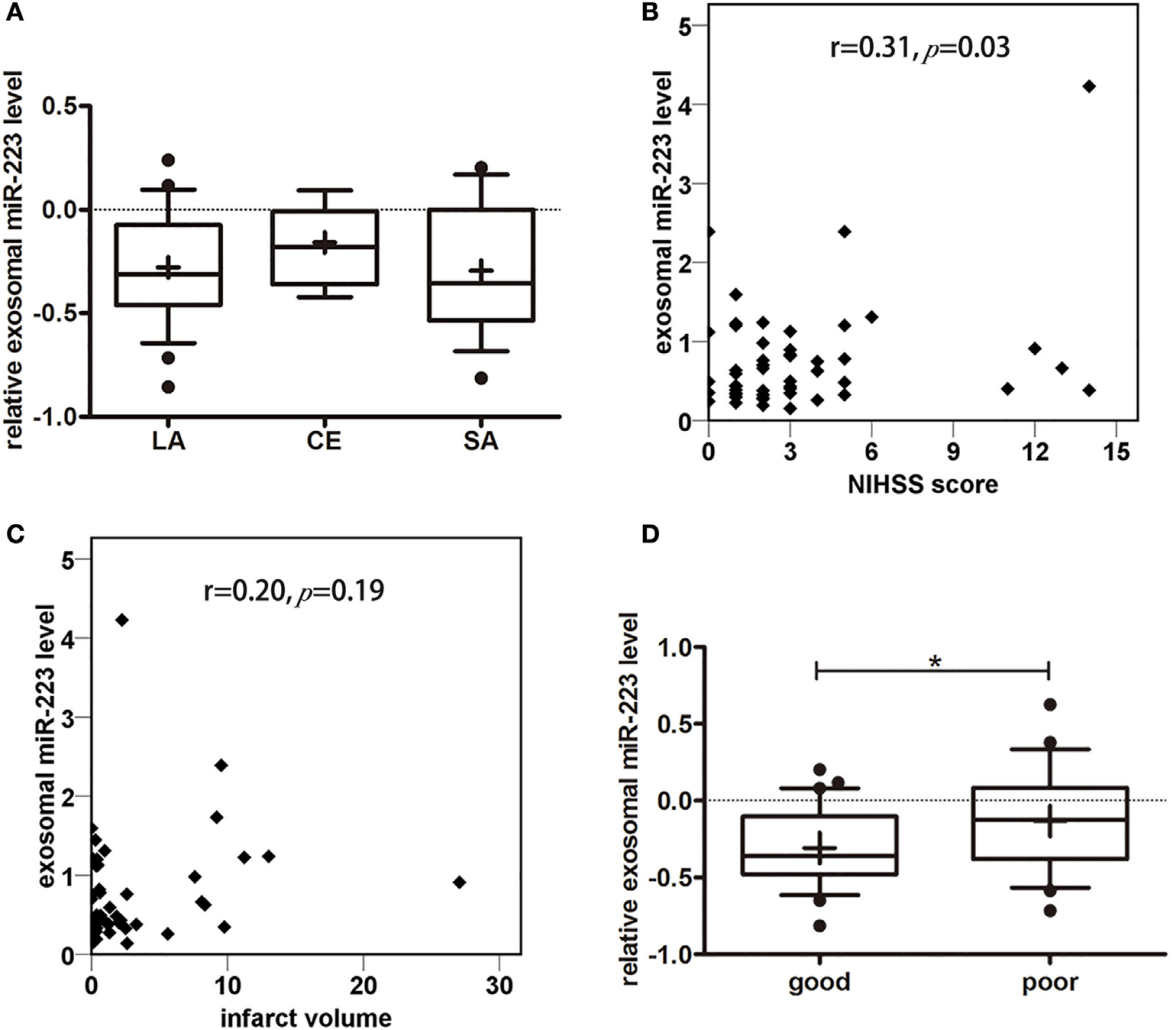
**Correlation analysis between miR-223 and clinical information**. **(A)** Serum exosomal miR-223 expression of stroke patients between different Trial of Org 10172 in Acute Stroke Treatment (TOAST) subtypes. Exosomal miR-223 level of different TOAST subtypes were compared with one-way ANOVA analysis. The *y*-axis indicated miR-223 expression levels by log10 change. Box plots showed the expression of exosomal miR-223 in different TOAST subtypes. Data are median (25% percentile, 75% percentile), *n* = 26 in LA, *n* = 7 in CE, *n* = 17 in SA. LA, large artery atherosclerotic stroke; CE, cardioembolism; SA, small artery stroke. **(B)** The correlation between exosomal miR-223 level and NIHSS score of stroke patients. The correlations between exosomal miR-223 level and NIHSS score was estimated by Pearson’s correlation test (*r* = 0.31, *p* = 0.03). The *y*-axis indicated miR-223 expression levels. NIHSS, National Institutes of Health Stroke Scale. **(C)** The correlation between exosomal miR-223 level and infarct volume. The correlations between exosomal miR-223 level and infarct volume was estimated by Pearson correlation test (*r* = 0.20, *p* = 0.19). The *y*-axis indicated miR-223 expression levels. **(D)** The correlation between exosomal miR-223 level and prognosis of stroke patients. The difference of exosomal miR-223 level between good prognosis patients [modified Rankin Scale (mRS) ≤2] and poor prognosis patients (mRS > 2) were compared with Student’s *t*-test. Box plots showed the expression of exosomal miR-223 in patients and control. The y-axis indicated miR-223 expression levels by log10 change. *p < 0.05, good prognosis patients vs. poor prognosis patients. Data are median (25% percentile, 75% percentile), *n* = 30 in good prognosis group, *n* = 20 in poor prognosis group.

To explore the relationship between circulating exosomal miR-223 and stroke subtypes, we compared exosomal miR-223 expression in stroke patients with different TOAST subtypes (Figure 4A). There was no difference in exosomal miR-223 level among TOAST subtypes, for example, LA (−0.28 ± 0.27, *n* = 26), cardioembolism (−0.16 ± 0.19, *n* = 7), and SA (−0.29 ± 0.31, *n* = 17, *p* = 0.53). Patients who were classified to cardioembolism seemed to have a higher level of exosomal miR-223 but needed further verification with larger sample. To examine the association between circulating exosomal miR-223 and NIHSS score, the Pearson correlation test was performed. Positive correlation was found between the exosomal miR-223 expression and NIHSS score (Figure 4B, *r* = 0.31, *p* = 0.03). As to the relationship between circulating exosomal miR-223 and infarct volume, no significant correlation was found in our study (Figure 4C, *r* = 0.20, *p* = 0.19). To evaluate the relationship between circulating exosomal miR-223 and 3-month prognosis, we compared the exosomal miR-223 level in stroke patients with good outcomes (mRS ≤ 2) and those with poor outcomes (mRS > 2). Stroke patients who had poor outcomes were shown to have a greater expression of exosomal miR-223 than patients who had good outcomes (Figure 4D, −0.1 ± 0.3 vs. −0.3 ± 0.3, *p* = 0.036).

## Discussion

In the present study, we demonstrated that (1) the level of miR-223 in circulating exosomes was elevated after onset of acute ischemic stroke; (2) exosomal miR-223 expression was positively correlated to NIHSS score; (3) stroke patients with poor outcomes inclined to have a greater exosomal miR-223 expression. Therefore, increased exosomal miRNA-223 is associated with acute ischemic stroke.

Abundant studies have demonstrated the extraordinary value of exosomes as strong weapon to diagnose and treat various diseases especially cancer (27–30). As to ischemic disease, previous studies largely focused on exploring the protection role of stem cell-derived exosomes after ischemic injury. Exosomes secreted by stem cells like cardiac progenitor cells, mesenchymal stem cells, and embryonic stem cells alleviated ischemia–reperfusion injury by delivering functional protein and RNAs (31–34). Chopp and colleagues found that systemic administration of mesenchymal stem cell-generated exosomes post stroke onset improved functional recovery with enhanced neurogenesis and angiogenesis (35). This protective role was likely mediated by miR-133 enriched exosomes transferring from mesenchymal stem cells to neurons and astrocytes (36).

So far there are few clinical studies about exosomes with ischemic vascular disease in patients (37, 38). Here, we first reported the existence of exosomes in human circulating blood with acute ischemic stroke. Exosomal miRNAs, compared to total cellular miRNAs or free miRNAs, are considered as more sensitive and specific biomarker since exosomes are secreted into extracellular space in a selective method (39–42). Chen detected the expression of circulating miR-126 and circulating exosomal miR-126 in transient middle cerebral artery occlusion rats. Exosomal miR-126 decreased significantly 3 h post stroke while circulating miR-126 remained unchanged (40). Our study further explored the possibility of utilizing exosomal miRNAs as novel stroke biomarkers among patients. There was a rise on exosomal miR-223 level post stroke, while no alteration in exosomal miR-21 and exosomal miR-145 level (unpublished data). miR-21, miR-145, and miR-223 are the most well-studied miRNAs that are related to hypoxia or ischemia regulation (13, 43–46).

Previous animal experiments revealed miR-223 as a double-edged sword in functional repair after ischemic injury (47–49). One clinical research also showed the same tendency of miR-223 expression in stroke patients. But the relationship between miR-223 level and NIHSS score was on the contrary to our study (12). The difference might be caused by different sources of RNA sample. The mean NIHSS score of stroke patients in our study was relatively low, indicating that exosomal miR-223 is sensitive for mild stroke. In the prospective population-based Bruneck study, reduced miR-223 and other four miRNAs levels in plasma antedated the manifestation of DM (50). A recent retrospective trail reported that platelet and plasma miR-223 expression were lower in DM patients with ischemic stroke than in DM only patients (51). The study also pointed that platelet miR-223 levels were correlated with plasma miR-223 level, but not with miR-223 level in leukocytes. In our study, we found no difference in serum exosomal miR-223 expression between participants with and without DM, or stroke patients with and without DM. Whether miR-223 expression was a risk factor for stroke in DM patients requires more research. Perhaps different sources of miR-223 play different role in stroke pathogenesis. miR-223 was reported to be elevated in the serum of patients with acute myocardial infarction and angina pectoris (52). But there is no hint whether the alteration is sustained in the long run. Our stroke patients did not contain patients with acute coronary syndrome since those patients would likely be sent to the Department of Cardiology.

Our study demonstrated that stroke patients with poor outcomes turned to have a greater expression of exosomal miR-223 (53). Future cohort study can be conducted to testify the association between higher miR-223 expression and lower stroke occurrence. Blocking the secretion and uptake of exosomes or regulating exosomal miR-223 expression might be necessary to exam the exact role of exosomal miR-223 in stroke recovery.

Exosomes, representing a novel approach to carry bioactive substances, provides a bright prospect in treating central nervous system diseases including stroke (54). On the one hand, these nano-sized particles can serve as efficient drug delivering system since they can cross blood brain barrier without much degradation (55). On the other hand, exosomes derived from stem cells may minimize adverse effects of administrating cells such as cell embolism and excessive cell differentiation (56). Endogenous exosomes after hypoxia precondition can also protect the tissue from ischemia–reperfusion injury (57). Hence, stem cell-derived exosomes or genetically modified exosomes are potential stroke treatment.

There are limitations of our study. The sample size was relatively small that we failed to further explore the value of using exosomal miR-223 to differentiate stroke causes. Future study with larger sample is needed. Besides, the blood sample was obtained within 72 h after stroke onset. Perhaps, this is relatively broad time range to detect the alteration of exosomal miR-223 level. It is more rational to collect the sample from the same patient dynamically at several earlier times such as 1, 3, 6, 12, 24, and 48 h. The correlation between exosomal miR-223 and NIHSS score was relatively weak; thus, it needs further investigation.

## Conclusion

This study showed that increased circulating exosomal miR-223 is associated with acute ischemic stroke occurrence, stroke severity, and short-term outcome. The clinical value of circulating exosomal miR-223 should be further assessed with large sample research. Functional studies on the role of exosomal miR-223 and its target in ischemic pathogenesis are also essential. We believe exosomal miR-223 has potential as a biomarker and a therapeutic target for ischemic stroke.

## Author Contributions

YC and YS designed the work, acquired and interpreted the data, and drafted the manuscript. JH, MQ, YZ, and JG collected and analyzed the clinical data, discussed the results, and participated in the final version. ZZ, JL, and GYY generated the conception of this work, analyzed and interpreted the data, and revised the manuscript. All the authors read and approved the final manuscript.

## Conflict of Interest Statement

The authors declare that the research was conducted in the absence of any commercial or financial relationships that could be construed as a potential conflict of interest.

## Abbreviations

CE, cardioembolism; DM, diabetes mellitus; LA, large artery atherosclerotic stroke; LACI, lacunar infarct; MCAO, middle cerebral artery occlusion; microRNA, miRNA; miR-223, microRNA-223; mRS, modified Rankin Scale; NIHSS, National Institutes of Health Stroke Scale; OCSP, Oxfordshire Community Stroke Project; PACI, partial anterior circulation infarct; POCI, posterior circulation infarct; ROC, receiver operator characteristic; TACI, total anterior circulation infarct; TEM, transmission electron microscopy; TOAST, Trial of Org 10172 in Acute Stroke Treatment.

## References

[1] Feigin VL Forouzanfar MH Krishnamurthi R Mensah GA Connor M Bennett DA Global and regional burden of stroke during 1990–2010: findings from the Global Burden of Disease Study 2010. Lancet (2014) 383(9913):245–55. 10.1016/s0140-6736(13)61953-4 24449944 PMC4181600

[2] Hijazi Z Lindback J Alexander JH Hanna M Held C Hylek EM The ABC (age, biomarkers, clinical history) stroke risk score: a biomarker-based risk score for predicting stroke in atrial fibrillation. Eur Heart J (2016) 37(20):1582–90. 10.1093/eurheartj/ehw054 26920728 PMC4875560

[3] Raman K O’Donnell MJ Czlonkowska A Duarte YC Lopez-Jaramillo P Penaherrera E Peripheral blood MCEMP1 gene expression as a biomarker for stroke prognosis. Stroke (2016) 47(3):652–8. 10.1161/STROKEAHA.115.011854 26846866

[4] Vijayan M Reddy PH. Peripheral biomarkers of stroke: focus on circulatory microRNAs. Biochim Biophys Acta (2016) 1862(10):1984–93. 10.1016/j.bbadis.2016.08.003 27503360 PMC5343760

[5] Lukiw WJ Pogue AI. Induction of specific micro RNA (miRNA) species by ROSgenerating metal sulfates in primary human brain cells. J Inorg Biochem (2007) 101(9):1265–9. 10.1016/j.jinorgbio.2007.06.004 17629564 PMC2080079

[6] Caporali A Emanueli C. MicroRNA regulation in angiogenesis. Vascul Pharmacol (2011) 55(4):79–86. 10.1016/j.vph.2011.06.006 21777698

[7] Baczyńska D Michałowska D Witkiewicz W. The role of microRNA in ischemic diseases – impact on the regulation of inflammatory, apoptosis and angiogenesis processes. Przegl Lek (2013) 70(3):135–42. 24003668

[8] Ksiazek-Winiarek DJ Kacperska MJ Glabinski A. MicroRNAs as novel regulators of neuroinflammation. Mediators Inflamm (2013) 2013:172351. 10.1155/2013/172351 23983402 PMC3745967

[9] Jickling GC Xu H Stamova B Ander BP Zhan X Tian Y Signatures of cardioembolic and large-vessel ischemic stroke. Ann Neurol (2010) 68(5):681–92. 10.1002/ana.22187 21031583 PMC2967466

[10] Tsai PC Liao YC Wang YS Lin HF Lin RT Juo SH. Serum microRNA-21 and microRNA-221 as potential biomarkers for cerebrovascular disease. J Vasc Res (2013) 50(4):346–54. 10.1159/000351767 23860376

[11] Zeng L Liu J Wang Y Wang L Weng S Chen S Cocktail blood biomarkers: prediction of clinical outcomes in patients with acute ischemic stroke. Eur Neurol (2013) 69(2):68–75. 10.1159/000342896 23154383

[12] Wang Y Zhang Y Huang J Chen X Gu X Wang Y Increase of circulating miR-223 and insulin-like growth factor-1 is associated with the pathogenesis of acute ischemic stroke in patients. BMC Neurol (2014) 14:77. 10.1186/1471-2377-14-77 24708646 PMC4234389

[13] Zhou J Zhang J. Identification of miRNA-21 and miRNA-24 in plasma as potential early stage markers of acute cerebral infarction. Mol Med Rep (2014) 10(2):971–6. 10.3892/mmr.2014.2245 24841240

[14] Huang S Zhao J Huang D Zhuo L Liao S Jiang Z. Serum miR-132 is a risk marker of post-stroke cognitive impairment. Neurosci Lett (2016) 615:102–6. 10.1016/j.neulet.2016.01.028 26806865

[15] Valadi H Ekstrom K Bossios A Sjostrand M Lee JJ Lotvall JO. Exosome-mediated transfer of mRNAs and microRNAs is a novel mechanism of genetic exchange between cells. Nat Cell Biol (2007) 9(6):654–9. 10.1038/ncb1596 17486113

[16] Chen TS Lai RC Lee MM Choo AB Lee CN Lim SK. Mesenchymal stem cell secretes microparticles enriched in pre-microRNAs. Nucleic Acids Res (2010) 38(1):215–24. 10.1093/nar/gkp857 19850715 PMC2800221

[17] Cheng Y Wang X Yang J Duan X Yao Y Shi X A translational study of urine miRNAs in acute myocardial infarction. J Mol Cell Cardiol (2012) 53(5):668–76. 10.1016/j.yjmcc.2012.08.010 22921780 PMC4492106

[18] Cazzoli R Buttitta F Di Nicola M Malatesta S Marchetti A Rom WN microRNAs derived from circulating exosomes as noninvasive biomarkers for screening and diagnosing lung cancer. J Thorac Oncol (2013) 8:1156–62. 10.1097/JTO.0b013e318299ac32 23945385 PMC4123222

[19] Huang X Yuan T Liang M Du M Xia S Dittmar R Exosomal miR-1290 and miR-375 as prognostic markers in castration-resistant prostate cancer. Eur Urol (2015) 67(1):33–41. 10.1016/j.eururo.2014.07.035 25129854 PMC4252606

[20] Yang Q Diamond MP Al-Hendy A. The emerging role of extracellular vesicle-derived miRNAs. J Clin Epigenet (2016) 2(1):1–16. 27099870 PMC4834835

[21] Van Giau V An SS. Emergence of exosomal miRNAs as a diagnostic biomarker for Alzheimer’s disease. J Neurol Sci (2016) 360:141–52. 10.1016/j.jns.2015.12.005 26723991

[22] Gui Y Liu H Zhang L Lv W Hu X. Altered microRNA profiles in cerebrospinal fluid exosome in Parkinson disease and Alzheimer disease. Oncotarget (2016) 6(35):37043–53. 10.18632/oncotarget.6158 26497684 PMC4741914

[23] Chevillet JR Kang Q Ruf IK Briggs HA Vojtech LN Hughes SM Quantitative and stoichiometric analysis of the microRNA content of exosomes. Proc Natl Acad Sci U S A (2014) 111(41):14888–93. 10.1073/pnas.1408301111 25267620 PMC4205618

[24] Raposo G Nijman HW Stoorvogel W Liejendekker R Harding CV Melief CJ B lymphocytes secrete antigen-presenting vesicles. J Exp Med (1996) 183(3):1161–72. 10.1016/j.ejmhg.2015.03.006 8642258 PMC2192324

[25] Colombo M Raposo G Thery C. Biogenesis, secretion, and intercellular interactions of exosomes and other extracellular vesicles. Annu Rev Cell Dev Biol (2014) 30:255–89. 10.1146/annurev-cellbio-101512-122326 25288114

[26] Colombo M Moita C van Niel G Kowal J Vigneron J Benaroch P Analysis of ESCRT functions in exosome biogenesis, composition and secretion highlights the heterogeneity of extracellular vesicles. J Cell Sci (2013) 126:5553–65. 10.1242/jcs.128868 24105262

[27] Fong MY Zhou W Liu L Alontaga AY Chandra M Ashby J Breast-cancer-secreted miR-122 reprograms glucose metabolism in premetastatic niche to promote metastasis. Nat Cell Biol (2015) 17(2):183–94. 10.1038/ncb3094 25621950 PMC4380143

[28] Hoshino A Costa-Silva B Shen TL Rodrigues G Hashimoto A Tesic Mark M Tumour exosome integrins determine organotropic metastasis. Nature (2015) 527(7578):329–35. 10.1038/nature15756 26524530 PMC4788391

[29] Melo SA Luecke LB Kahlert C Fernandez AF Gammon ST Kaye J Glypican-1 identifies cancer exosomes and detects early pancreatic cancer. Nature (2015) 523(7559):177–82. 10.1038/nature14581 26106858 PMC4825698

[30] Munagala R Aqil F Gupta RC. Exosomal miRNAs as biomarkers of recurrent lung cancer. Tumour Biol (2016) 37(8):10703–14. 10.1007/s13277-016-4939-8 26867772

[31] Xin H Li Y Chopp M. Exosomes/miRNAs as mediating cell-based therapy of stroke. Front Cell Neurosci (2014) 8:377. 10.3389/fncel.2014.00377 25426026 PMC4226157

[32] Feng Y Huang W Wani M Yu X Ashraf M. Ischemic preconditioning potentiates the protective effect of stem cells through secretion of exosomes by targeting Mecp2 via miR-22. PLoS One (2014) 9(2):e88685. 10.1371/journal.pone.0088685.g001 24558412 PMC3928277

[33] Gray WD French KM Ghosh-Choudhary S Maxwell JT Brown ME Platt MO Identification of therapeutic covariant microRNA clusters in hypoxia-treated cardiac progenitor cell exosomes using systems biology. Circ Res (2015) 116(2):255–63. 10.1161/CIRCRESAHA.116.304360 25344555 PMC4338016

[34] Khan M Nickoloff E Abramova T Johnson J Verma SK Krishnamurthy P Embryonic stem cell-derived exosomes promote endogenous repair mechanisms and enhance cardiac function following myocardial infarction. Circ Res (2015) 117(1):52–64. 10.1161/CIRCRESAHA.117.305990 25904597 PMC4482130

[35] Xin H Li Y Cui Y Yang JJ Zhang ZG Chopp M. Systemic administration of exosomes released from mesenchymal stromal cells promote functional recovery and neurovascular plasticity after stroke in rats. J Cereb Blood Flow Metab (2013) 33(11):1711–5. 10.1038/jcbfm.2013.152 23963371 PMC3824189

[36] Xin H Li Y Buller B Katakowski M Zhang Y Wang X Exosome-mediated transfer of miR-133b from multipotent mesenchymal stromal cells to neural cells contributes to neurite outgrowth. Stem Cells (2012) 30(7):1556–64. 10.1002/stem.1129 22605481 PMC3495063

[37] Kuwabara Y Ono K Horie T Nishi H Nagao K Kinoshita M Increased microRNA-1 and microRNA-133a levels in serum of patients with cardiovascular disease indicate myocardial damage. Circ Cardiovasc Genet (2011) 4:446–54. 10.1161/CIRCGENETICS.110.958975 21642241

[38] Li J Rohailla S Gelber N Rutka J Sabah N Gladstone RA MicroRNA-144 is a circulating effector of remote ischemic preconditioning. Basic Res Cardiol (2014) 109(5):423–37. 10.1007/s00395-014-0429-610.1007/s00395-014-0423-z 25060662

[39] Squadrito ML Baer C Burdet F Maderna C Gilfillan GD Lyle R Endogenous RNAs modulate microRNA sorting to exosomes and transfer to acceptor cells. Cell Rep (2014) 8(5):1432–46. 10.1016/j.celrep.2014.07.035 25159140

[40] Chen F Du Y Esposito E Liu Y Guo S Wang X Effects of focal cerebral ischemia on exosomal versus serum miR126. Transl Stroke Res (2015) 6(6):478–84. 10.1007/s12975-015-0429-3 26449616

[41] Hazan-Halevy I Rosenblum D Weinstein S Bairey O Raanani P Peer D. Cell-specific uptake of mantle cell lymphoma-derived exosomes by malignant and non-malignant B-lymphocytes. Cancer Lett (2015) 364(1):59–69. 10.1016/j.canlet.2015.04.026 25933830 PMC4490183

[42] Sohn W Kim J Kang SH Yang SR Cho J-Y Cho HC Serum exosomal microRNAs as novel biomarkers for hepatocellular carcinoma. Exp Mol Med (2015) 47(9):e184. 10.1038/emm.2015.68 26380927 PMC4650928

[43] Taibi F Metzinger-Le Meuth V Massy ZA Metzinger L. miR-223: an inflammatory oncomiR enters the cardiovascular field. Biochim Biophys Acta (2014) 1842(7):1001–9. 10.1016/j.bbadis.2014.03.005 24657505

[44] Xu X Kriegel AJ Jiao X Liu H Bai X Olson J miR-21 in ischemia/reperfusion injury: a double-edged sword? Physiol Genomics (2014) 46(21):789–97. 10.1152/physiolgenomics.00020.2014 25159851 PMC4280148

[45] Jia L Hao F Wang W Qu Y. Circulating miR-145 is associated with plasma high-sensitivity C-reactive protein in acute ischemic stroke patients. Cell Biochem Funct (2015) 33(5):314–9. 10.1002/cbf.3116 26096228

[46] Zhao W Zhao SP Zhao YH MicroRNA-143/-145 in cardiovascular diseases. Biomed Res Int (2015) 2015:531740 10.1155/2015/531740 26221598 PMC4499377

[47] Harraz MM Eacker SM Wang X Dawson TM Dawson VL. MicroRNA-223 is neuroprotective by targeting glutamate receptors. Proc Natl Acad Sci U S A (2011) 109:18962–189671. 10.1073/pnas.1121288109 23112146 PMC3503176

[48] Duan X Zhan Q Song B Zeng S Zhou J Long Y Detection of platelet microRNA expression in patients with diabetes mellitus with or without ischemic stroke. J Diabetes Complications (2014) 28(5):705–10. 10.1016/j.jdiacomp.2014.04.012 24908639

[49] Dai GH Ma PZ Song XB Liu N Zhang T Wu B. MicroRNA-223-3p inhibits the angiogenesis of ischemic cardiac microvascular endothelial cells via affecting RPS6KB1/hif-1a signal pathway. PLoS One (2014) 9(10):e108468. 10.1371/journal.pone.0108468.g001 25313822 PMC4196764

[50] Zampetaki A Kiechl S Drozdov I Willeit P Mayr U Prokopi M Plasma microRNA profiling reveals loss of endothelial miR-126 and other microRNAs in type 2 diabetes. Circ Res (2010) 107(6):810–7. 10.1161/CIRCRESAHA.110.226357 20651284

[51] Yang S Zhao J Chen Y Lei M Biomarkers associated with ischemic stroke in diabetes mellitus patients. Cardiovasc Toxicol (2016) 16(3):213–22. 10.1007/s12012-015-9329-8 26175178

[52] Li C Fang Z Jiang T Zhang Q Liu C Zhang C Serum microRNAs profile from genome-wide serves as a fingerprint for diagnosis of acute myocardial infarction and angina pectoris. BMC Med Genomics (2013) 6:16. 10.1186/1755-8794-6-16 23641832 PMC3655858

[53] Tan KS Armugam A Sepramaniam S Lim KY Setyowati KD Wang CW Expression profile of microRNAs in young stroke patients. PLoS One (2009) 4(11):e7689. 10.1371/journal.pone.0007689.g001 19888324 PMC2765616

[54] Zhang ZG Chopp M Exosomes in stroke pathogenesis and therapy. J Clin Invest (2016) 126(4):1190–7. 10.1172/JCI81133 27035810 PMC4811130

[55] Johnsen KB Gudbergsson JM Skov MN Pilgaard L Moos T Duroux M A comprehensive overview of exosomes as drug delivery vehicles – endogenous nanocarriers for targeted cancer therapy. Biochim Biophys Acta (2014) 1845:75–87. 10.1016/j.bbcan.2014.04.005 24747178

[56] Lai RC Chen TS Lim SK. Mesenchymal stem cell exosome: a novel stem cell-based therapy for cardiovascular disease. Regen Med (2012) 6(4):481–92. 10.2217/rme.11.35 21749206

[57] Vicencio JM Yellon DM Sivaraman V Das D Boi-Doku C Arjun S Plasma exosomes protect the myocardium from ischemia-reperfusion injury. J Am Coll Cardiol (2015) 65(15):1525–36. 10.1016/j.jacc.2015.02.026 25881934

